# Exopolysaccharide of *Enterococcus faecium* L15 promotes the osteogenic differentiation of human dental pulp stem cells via p38 MAPK pathway

**DOI:** 10.1186/s13287-022-03151-0

**Published:** 2022-09-02

**Authors:** Hyewon Kim, Naeun Oh, Mijin Kwon, Oh-Hee Kwon, Seockmo Ku, Jeongmin Seo, Sangho Roh

**Affiliations:** 1 Cellular Reprogramming and Embryo Biotechnology Laboratory, Dental Research Institute, Seoul National University School of Dentistry, Seoul, 08826 Korea; 2 Biomedical Research Institute, NeoRegen Biotech Co., Ltd., Gyeonggi-do, 16641 Korea; 3 Fermentation Science Program, School of Agriculture, College of Basic and Applied Sciences, Middle Tennessee State University, Murfreesboro, TN 37132 USA

**Keywords:** *Enterococcus faecium*, Exopolysaccharide, Dental pulp stem cell, Bone, Calvaria, p38 MAPK pathway

## Abstract

**Background:**

Bone has important functions in the body. Several researchers have reported that the polysaccharides and lipopolysaccharide derived from microbes can promote osteogenic differentiation of stem cells. *Enterococcus faecium,* a lactic acid bacterium (LAB), produces several bioactive metabolites and has been widely applied in the food and nutraceutical industries. The exopolysaccharide (EPS) from LAB has also been extensively examined for its postbiotic effects and for its in vivo and in vitro functionalities. However, studies on promoting bone differentiation using polysaccharides from LAB are lacking. Therefore, the purpose of this study was to investigate the effect of *E. faecium* L15 extract and EPS on osteogenic differentiation of human dental pulp stem cells (hDPSCs) and to identify the underlying mechanisms.

**Methods:**

hDPSCs were obtained from dental pulp tissue, and L15 extract and EPS were isolated from L15. Gene and protein expression of the osteogenic differentiation markers were analyzed with qPCR and western blotting and the possible signaling pathways were also investigated using western blotting. Osteogenic differentiation potential was examined by alkaline phosphatase (ALP) staining and alizarin red s (ARS) staining. In addition, osteogenic differentiation potential of L15 EPS was explored in ex vivo culture of neonate murine calvaria.

**Results:**

The calcium deposition and ALP activity were enhanced by addition of L15 extract or EPS. The expression levels of *RUNX2*, *ALP*, and *COL1A1* mRNA and the protein expression levels of RUNX2, ALP, and BMP4 were increased in hDPSCs treated with the L15 extract or EPS. The L15 EPS treatment enhanced phosphorylation of the p38 mitogen-activated protein kinase (MAPK). The L15 EPS-induced increases in RUNX2, ALP, and BMP4 expression were suppressed by the p38 MAPK inhibitor SB203580. The promoting effect of L15 EPS on osteogenic differentiation was not only seen in hDPSCs, but also in osteoblast precursors. ALP activity and the expression of *RUNX2*, *ALP*, and *COL1A1* increased in the L15 EPS-treated osteoblast precursors. In addition, L15 EPS increased bone thickness of neonate murine calvaria in ex vivo culture.

**Conclusions:**

The stimulatory effect of L15 extract and EPS on osteogenic differentiation occurred through the p38 MAPK pathway, and L15 EPS enhanced new bone formation in neonate murine calvaria. These data suggest that L15 EPS has therapeutic potential applicable to bone regeneration.

**Supplementary Information:**

The online version contains supplementary material available at 10.1186/s13287-022-03151-0.

## Background

Bone is a hard connective tissue of the body and serves to protect various organs, produce red and white blood cells, store minerals, and support the structure of the body [1]. Bone has the intrinsic capacity to repair itself, but in case of severe damage, it is difficult to self-repair [2]. Bone regeneration is a major challenge in reconstructive surgery [2]. Since stem cells have the capacity to self-renew and differentiate into various other types of cells, they are used for tissue regeneration after injury or disease [3, 4]. Dental pulp stem cells (DPSCs) are neural crest-derived mesenchymal stem cells (MSCs) that have the potential to differentiated into adipogenic, osteogenic, chondrogenic, and myogenic cells [5]. DPSCs can be isolated from the pulp tissue of the tooth by non-invasive methods [6, 7]. It has been reported that DPSCs have the potential to be used to cure some neurological, immunodeficiency disease, and cartilage diseases [8–10].

Various genera of lactic acid bacteria (LAB) (e.g., *Carnobacterium, Enterococcus, Lactobacillus, Lactococcus, Leuconostoc, Bifidobacterium, Oenococcus, Pediococcus, Streptococcus, Tetragenococcus, Vagococcus,* and *Weissella*) are widely used as potential probiotic strains in personal care and cosmetic products [11–13]. LAB are known to provide their hosts with several health benefits, including the promotion of intestinal and systemic immune system responses, improvement of intestinal mucosal barrier integrity, relief of chronic constipation, treatment of diarrhea, and modulation of gut microbiota [14–16]. Among LAB genera, *Enterococcus* spp., the gram-positive, facultative anaerobic bacteria are capable of survival under harsh environmental conditions [17] and are commonly found in traditional fermented foods. One spp., *Enterococcus faecium*, has antioxidant and anti-inflammatory properties and displays antimicrobial effects against pathogens, both in vitro and in vivo [18, 19]. Recently, products containing *E. faecium* have been commercially launched to relieve diarrhea, and some are being licensed in several European countries (e.g., Austria, Italy, and Switzerland) [20].

LAB produce long-chain bioactive carbohydrate polymers, known as exopolysaccharides (EPS) [13, 21], and some LAB-produced EPSs show anti-cancer, antioxidant, and cholesterol-lowering effects [22–24]. EPS from *E. coli* have also been shown to enhance osteogenic differentiation [25–28]. However, the practical application of *E. coli* in the nutraceutical and/or pharmaceutical industries can be hindered by issues arising in the marketing and consumer safety of products containing nonedible microorganisms [29]. Moreover, despite the functional advantages of postbiotic molecules, investigations of these substances are often limited by their unknown molecular mechanisms of action. Few studies have reported the effects of LAB on osteogenic differentiation [30–32] therefore, the aim of this study was to investigate the effects of *E. faecium* L15 extracts and their EPS on osteogenic differentiation of hDPSCs and the underlying mechanisms.

## Methods

### Preparation of *E. faecium* L15 extract

The *E. faecium* L15 strain (KCTC13498BP, “L15”) was used for this study and was obtained from NeoRegen Biotech (Suwon, Gyeonggi-do, Korea). The L15 extract preparation process followed our previously published laboratory protocol [10]. This strain was originally isolated from a traditional Korean rice-fermented food containing flatfish. L15 was cultured in tryptic soy broth (TSB; Hardy Diagnostics, Santa Maria, CA, USA) and incubated for 18 h at 35 °C. The cultured L15 was harvested, washed three times in phosphate-buffered saline (PBS), and resuspended in double-deionized water (ddH_2_O). The washed L15 was sonicated (Sonics, Stratford, CT, USA) on ice for 30 min. To remove the cellular debris, it was centrifuged at 12,000×*g* for 10 min. The supernatant was passed through a 0.45 μm filter and frozen at − 80 ℃ overnight. It was then freeze-dried and reconstituted with PBS before use.

### Preparation of the *E. faecium* L15 EPS

The EPS obtained from L15 was purified using ethanol precipitation method [33]. L15 was isolated from the growth media by centrifugation at 10,000×*g* for 20 min. After centrifugation, the supernatant media was collected and added with a final concentration of 14% trichloroacetic acid to denature the protein and nucleic acid, and then incubated for 1 h at 37 °C Absolute ethanol was mixed with the L15 supernatant in the ratio of 2:1 and the solution was incubated at 4 °C for 1 day for aggregation. The precipitate was dissolved in ddH_2_O and dialyzed for 24–48 h at 4 °C to remove traces of protein. The precipitate was frozen at − 80 °C, freeze-dried, and reconstituted with PBS for use in the following experiments.

### Fractionation of EPS

Fractionation of the crude EPS was accomplished by size exclusion chromatography [34]. The purified crude EPS was applied to a HiLoad™ 16/600 Superdex 200 (GE Healthcare, Chicago, IL, USA) column equilibrated with PBS. Elution was performed at a flow rate of 1 ml min^−1^, and 2 ml fractions were collected using a fraction collector.

### Isolation and expansion of human dental pulp stem cells (hDPSCs)

This study was conducted in accordance with the Declaration of Helsinki, and the protocol was approved by the Institutional Review Board (IRB, number S-D20100005) at the Seoul National University School of Dentistry. Human maxillary central supernumerary teeth (*n* = 3) were extracted at the Dental Hospital of Seoul National University in accordance with the guidelines approved by the IRB. The hDPSC primary culture process followed our laboratory protocol [35]. The extracted teeth were briefly cut around the cemento–enamel junction using a cutting disk. The pulp tissue was exposed and gently separated from the crown. The pulp tissue was minced into 1 mm^2^ pieces with a scalpel blade and transferred into 12-well culture dishes. The cells were then grown in Minimum essential medium eagle—alpha modification (α-MEM; Hyclone Laboratories Inc., Logan, UT, USA) supplemented with 10% fetal bovine serum (FBS; Hyclone Laboratories Inc.) and incubated in a 37 °C incubator with 5% CO_2_. The culture media was replaced every three days. The cells from different donors were cultured separately.

### Characterization of hDPSCs by fluorescence-activated cell sorting (FACS)

FACS was performed to identify hDPSCs as described previously [10]. At passage 3 and 8, the hDPSCs were detached and resuspended in ice-cold PBS containing 5% FBS. The cells were incubated on ice for 30 min with monoclonal antibodies against CD10-fluorescein isothiocyanate (FITC), CD29-Alexa 488, CD44-FITC, CD73-FITC, CD90-FITC, CD105-FITC, CD14-allophycocyanin (APC), CD34-Alexa 647, CD45-APC, and CD31-APC. Analyses were performed using a FACSVerse (Becton Dickinson, Franklin Lakes, NJ, USA).

### Osteogenic differentiation of hDPSCs

hDPSCs were seeded at 15,000 cells/cm^2^ onto 12-well culture dishes in osteogenic differentiation media consisting of α-MEM (Hyclone Laboratories Inc.) supplemented with 10% FBS (Hyclone Laboratories Inc.), 100 nM dexamethasone (Sigma-Aldrich), 10 mM β-glycerophosphate (Sigma-Aldrich) and 0.05 mM ascorbic acid 2-phosphate (Sigma-Aldrich) [36]. The media was changed every 2 to 3 days and osteogenic differentiation was conducted for 28 days.

### Cell viability assay

Cell viability was determined using the EZ-Cytox kit (Daeil Lab Service, Seoul, Korea), based on the water-soluble tetrazolium salt (WST) method [10]. The hDPSCs were seeded in 96-well plates at a density of 1 × 10^4^ cells per well. Cells were cultured in osteogenic differentiation media with various concentrations of L15 extracts for 3 days and L15 EPS for 3 days and 7 days. WST solution was added to each well. The mixture was incubated for 30 min at 37 °C. The absorbance of each well was measured at 450 nm with an Emax Plus Microplate reader (Molecular Devices, Sunnyvale, CA, USA).

### Reverse transcription polymerase chain reaction (RT-PCR) and Real-time PCR

RT-PCR and real-time PCR were used to quantify gene expression [10]. Total RNA was extracted from pellets using PureLink™ RNA Mini kits (Life Technologies, Camarillo, CA, USA). The synthesis of cDNA was performed using M-MLV reverse transcriptase (Promega Corporation, Fitchburg, WI, USA) according to the manufacturer’s instructions. Real-time PCR was performed using SYBR Pre-mix Ex Taq™ II (Takara, Tokyo, Japan) and a 7500 Real-Time PCR System (Applied Biosystems, Carlsbad, CA, USA). The primers used are listed in Table 1. The PCR reaction was performed for 30 s at 95 °C, followed by 40 amplification cycles of 5 s at 95 °C and 34 s at 60 °C. The comparative C_T_ method was used to measure the level of expression. Glyceraldehyde 3-phosphate dehydrogenase (GAPDH) was used as a housekeeping gene for normalization.

**Table 1:** Primer sequences used for real time-PCR

Gene	Forward primer (5′-3′)	Reverse primer (5′-3′)	Reference
Human genes			
Runx2	CAGACCAGCAGCACTCCATA	TTCAATATGGTCGCCAAACA	[37]
COL1A1	GCGAGAGCATGACCGATGGA	GCGGATCTCGATCTCGTTGGA	[38]
ALP	GGGATAAAGCAGGTCTTGGGGTGC	CGCTTGGTCTCGCCAGTACTTGG	[39]
GAPDH	ACATGTTCCAATATGATTCC	TGGACTCCACGACGTACTCA	[40]
Mouse genes			
Runx2	Identical with human primers, see above	Identical with human primers, see above	[37]
COL1A1	Identical with human primers, see above	Identical with human primers, see above	[38]
ALP	CCAACTCTTTTGTGCCAGAGA	GGCTACATTGGTGTTGAGCTTTT	[41]
GAPDH	AGGTCGGTGTGAACGGATTTG	TGTAGACCATGTAGTTGAGGTCA	[42]

### Alizarin red S (ARS) staining

The osteogenic differentiation was evaluated by ARS staining to visualize calcium deposits [36]. The differentiated cells were fixed with 4% paraformaldehyde (PFA) and stained with alizarin red solution (Sigma-Aldrich) then photographed by a digital camera (Canon, Tokyo, Japan) and observed under an inverted microscope (EVOS™ XL Core Imaging System; Thermo Scientific™, Waltham, MA, USA).

### Alkaline phosphatase (ALP) staining

ALP staining was performed with StemAb Alkaline Phosphatase Staining Kit II (Reprocell, Beltsville, MD, USA) according to the manufacturer’s instructions [43]. Briefly, hDPSCs and mouse calvaria-derived osteoblasts were treated with or without L15 extract or L15 EPS for 7 days. Cells were fixed for 2 min using a fixation solution, washed two times with PBS, and incubated with ALP staining solution for 30 min. ALP staining solution was removed and the staining observed under an inverted microscope (EVOS™ XL Core Imaging System; Thermo Scientific™, Waltham, MA, USA).

### Western blot analysis

Cytoplasmic and nuclear proteins were extracted by NE-PER™ Nuclear and Cytoplasmic Extraction Reagents (Thermo Scientific™) with proteinase inhibitor (MedChemExpress, Monmouth Junction, NJ, USA) and phosphatase inhibitor (MedChemExpress) [44]. The cell lysates were separated with sodium dodecyl sulfate polyacrylamide gel electrophoresis and immunoblotted with the following indicated antibodies: bone morphogenetic protein-4 (BMP4; Cusabio Life Science, Wuhan, China), runx family transcription factor 2 (Runx2; Novus Biologicals, Centennial, Colorado, USA), ALP (R&D Systems, Minneapolis, MN, USA), extracellular signal-regulated kinase (ERK; Cell Signaling Technology), phospho-ERK (p-ERK; Cell Signaling Technology), c-Jun N-terminal kinase (JNK; Cell Signaling Technology), p-JNK (Cell Signaling Technology), p38 (Cell Signaling Technology), p-p38 (Cell Signaling Technology), β-catenin (Cell Signaling Technology), Lamin B1 (Santa Cruz Biotechnology, Dallas, TX, USA), and GAPDH (BioLegend, SanDiego, CA, USA). GAPDH was used as a housekeeping gene for normalization. Lamin B1 was used for normalization of nuclear β-catenin.

### Calvaria-derived osteoblast precursor isolation

Primary osteoblast precursors from mouse calvaria were isolated as described previously [45]. Briefly, calvaria were isolated from 4-day-old mice and digested with 0.25% trypsin and 0.2% collagenase at 37 °C for 30 min. Released cells were plated in a 100-mm dish, grown in α-MEM (Hyclone Laboratories Inc.) supplemented with 10% FBS (Hyclone Laboratories Inc.), and incubated in a 37 °C incubator with 5% CO_2_. After 3 days, adherent cells were used as osteoblast precursors. The osteoblast precursors were used for osteogenic differentiation with L15 EPS.

### Mouse calvarial organ culture and hematoxylin and eosin staining

Animal experiments were approved by the Institutional Animal Care and Use Committee of Seoul National University (IACUC, number SNU-200204–1). The calvaria from ICR mice at postnatal day 4 were cultured on a grid in a 12-well culture plate. Calvaria were cultured in hDPSC culture media with or without 10 μg/mL L15 EPS. The media was changed every 2 days, and calvaria were harvested on day 7. Calvaria were fixed in 4% PFA for 24 h, decalcified in 14% EDTA for 2 days. After decalcification, the calvaria were embedded in paraffin wax. The blocks were trimmed to a depth of 800 μm and sagittally sectioned to a 10 μm (Leica Microsystems, Wetzlar, Germany) thickness from the midline. The sectioned tissues were stained with hematoxylin and eosin (H&E), and bright field micrographs were captured under an Olympus BX50 microscope (Olympus, Tokyo, Japan). Bone thickness was measured using Image Pro software (Media Cybernetics Inc., Silver Spring, MD, USA) from sagittal sections obtained from a specific position [1 mm far from midline suture of calvaria] [46].

### Statistical analysis

Results are presented as mean ± S.D. Data were analyzed using a one-way analysis of variance (ANOVA) followed by Tukey post hoc test and Student’s *t*-test with GraphPad Prism V5.0 software (GraphPad Software, La Jolla, CA, USA). *p < 0.05, **p < 0.01, and ***p < 0.001 were defined as statistical significance.

## Results

### Characterization of human dental pulp stem cells (hDPSCs)

**Fig. 1:**
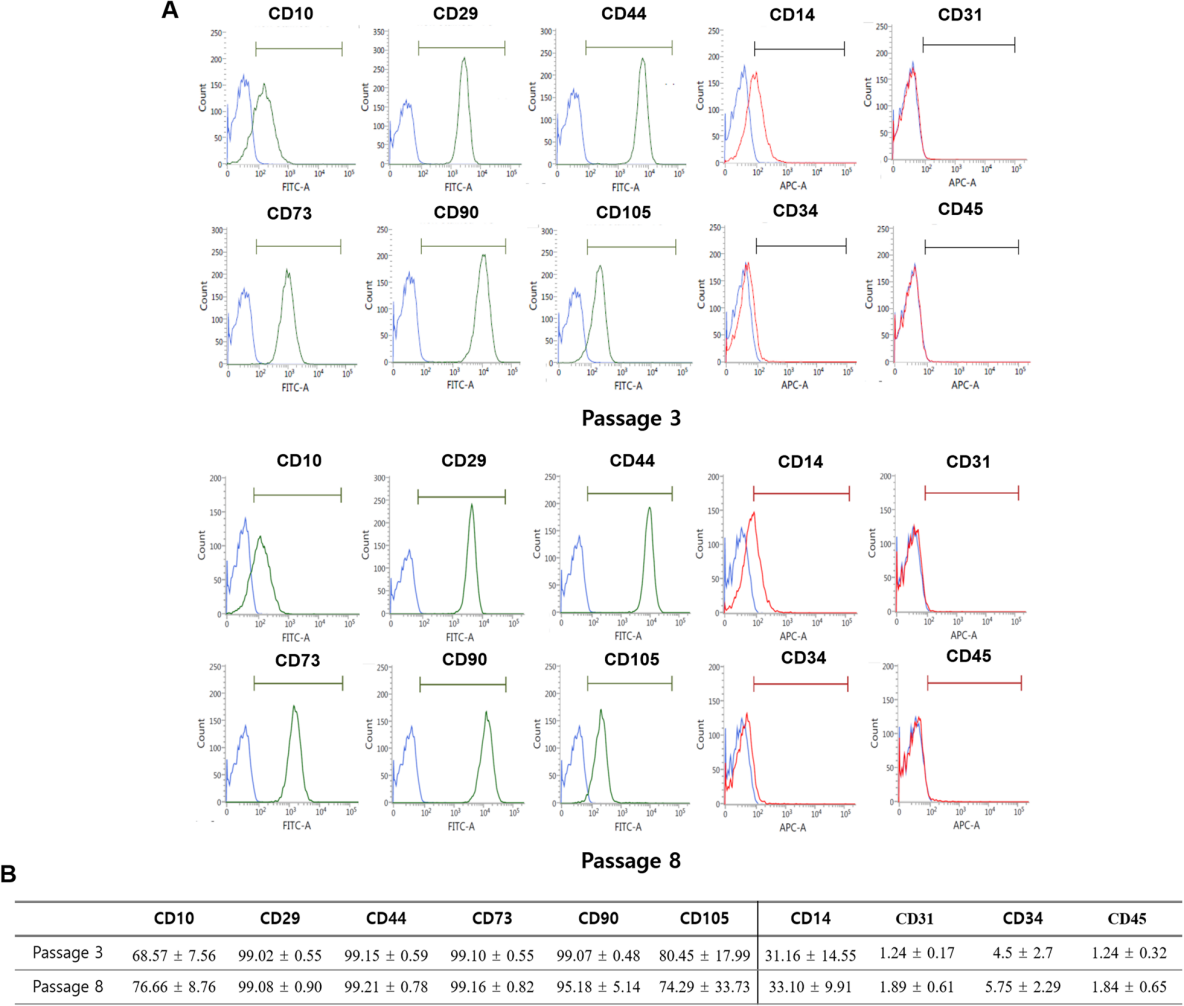
The hDPSCs characterization of passage 3 and passage 8 by fluorescence-activated cell sorting (FACS). **A** FACS analysis indicated the mesenchymal, hematopoietic, and endothelial stem cell marker expression of hDPSCs, passage 3 and passage 8. **B** Values are expressed as percentage. *N* = 3

To investigate the properties of hDPSCs, the cells were analyzed by fluorescence-activated cell sorting (FACS) (Fig. 1A). At passage 3 of hDPSCs, the cells expressed high levels of MSC markers, but low levels of hematopoietic and endothelial stem cell markers (Fig. 1B). At passage 8 of hDPSCs, the ratio of surface marker expression was similar to that of passage 3 (Fig. 1B). Therefore, passage 3–8 cells were used for osteogenic differentiation.

### Effect of L15 extract on hDPSC viability

**Fig. 2:**
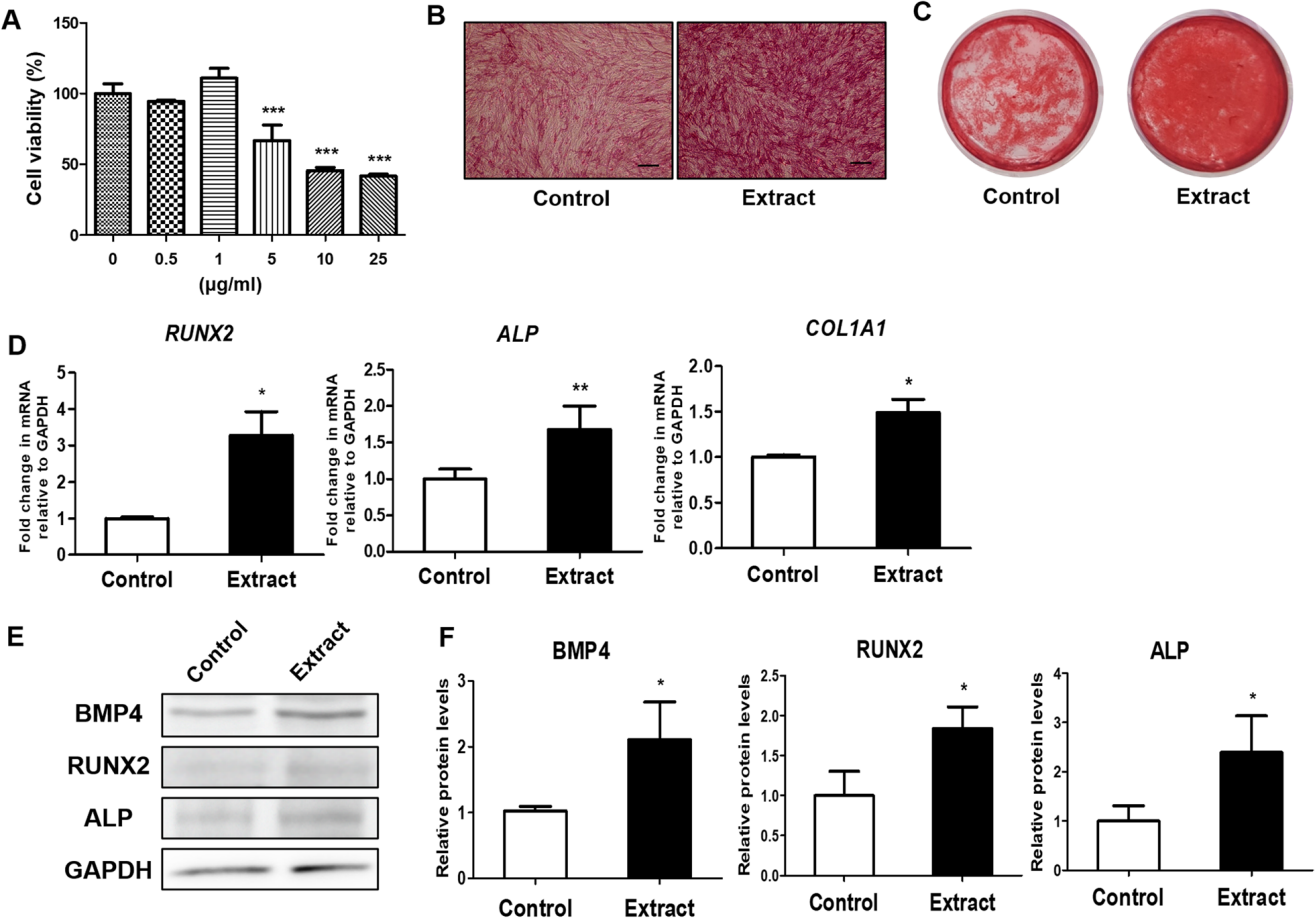
L15 extract promotes osteogenic differentiation of hDPSCs. **A** Viability of hDPSCs on exposure to various doses of L15 extract. **B** Bright field micrographs showing ALP staining of hDPSCs with or without L15 extract (EVOS™ XL Core Imaging System; Scale bar = 200 μm). **C** Calcium deposition of hDPSCs with or without L15 extract by ARS staining. The expression of osteogenic differentiation marker genes (*RUNX2, ALP,* and *COL1A1*) were analyzed by real time-PCR (**D**). *GAPDH* was used for normalization. **E** The expression of osteogenic differentiation marker proteins (BMP4, RUNX2, and ALP) expression. **F** Quantitative analysis of intensity in **E** relative to GAPDH. Error bars represent standard deviation of the mean. *p < 0.05, **p < 0.01, ***p < 0.001. *N* = 3

As shown in Fig. 2A, hDPSC viability was significantly decreased by treatments of 5 μg/mL or more (*p* < 0.001). This suggested that an L15 extract concentration of 1 μg/mL was non-toxic to cells, and this concentration was used for subsequent osteogenic differentiation.

### L15 extract facilitates osteogenic differentiation of hDPSCs

hDPSCs were cultured with L15 extract for 7 days, after which ALP staining was performed. Darker ALP staining was observed in the L15 extract treatment group compared to the control group (Fig. 2B). Calcium deposition was examined by ARS staining after osteogenic differentiation for 28 days. Enhanced calcium deposition was observed in the L15 extract treatment group compared to the control group (Fig. 2C). The expression levels of *RUNX2*, alkaline phosphatase (*ALP*), and collagen type I (*COL1A1*), which are osteogenesis-related genes, were examined by real-time PCR. The expression levels of *RUNX2*, *ALP*, and *COL1A1* were significantly increased after L15 extract treatment (Fig. 2D; *p* < 0.05*, p* < 0.01). The effect of L15 extract on the levels of osteogenesis-related proteins (i.e., bone morphogenetic protein 4 (BMP4), RUNX2, and ALP) were verified by western blot (Fig. 2E). BMP4, RUNX2, and ALP expression were significantly increased in the L15 extract treatment group compared to the control group (Fig. 2F).

### Screening for the optimal L15 EPS concentration

**Fig. 3:**
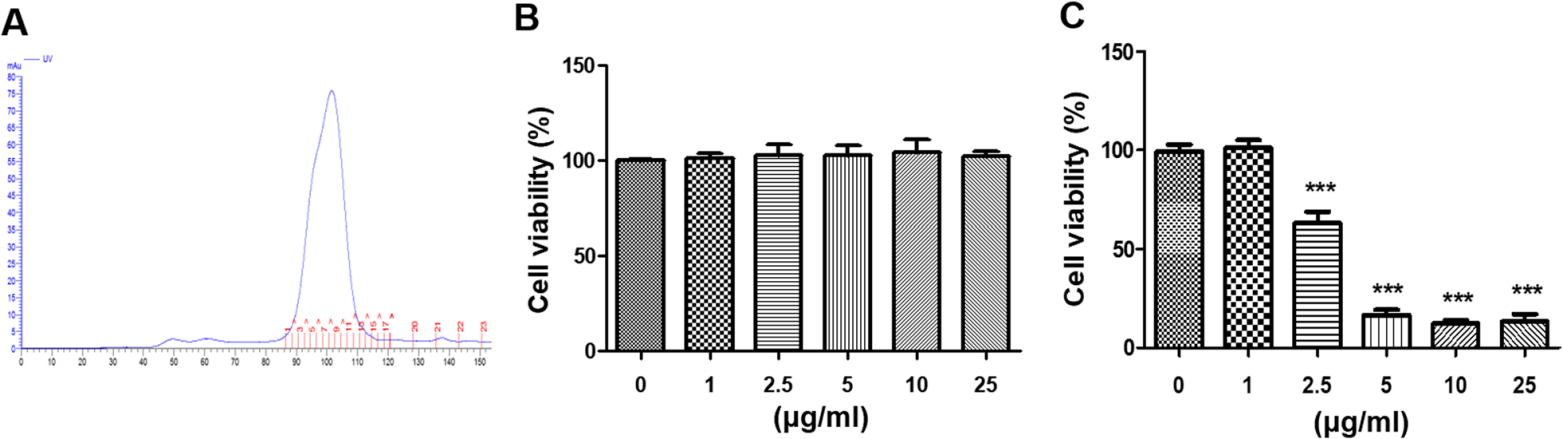
Toxicity of L15 EPS on hDPSCs. **A** Size-exclusion chromatogram of L15 EPS. **B** hDPSC viability on exposure to different doses of L15 EPS with osteogenic differentiation medium for 3 days and **C** 7 days. ***p < 0.001. Error bars represent mean ± S.D. Scale bar = 200 μm. *N* = 3

The purified EPS of L15 was fractionated using size exclusion chromatography. As shown in Fig. 3A, L15 EPS appeared as a single peak, which corresponded to a low molecular weight fraction. To determine the effect of L15 EPS on hDPSCs viability, a water-soluble tetrazolium salt (WST) assay was performed [47]. Six concentrations of L15 EPS (0, 1, 2.5, 5, 10, and 25 μg/ml) were treated for 3 days. There was no significant difference between the groups (Fig. 3B). The same concentrations of L15 EPS were treated with osteogenic differentiation media for 7 days. hDPSCs viability was significantly decreased by treatments of 2.5 μg/mL or more of L15 EPS (Fig. 3C). This result suggested that the continuous treatment of 2.5 μg/ml L15 EPS was toxic to the osteogenic differentiation of hDPSCs, and 1 μg/ml L15 EPS was used for subsequent experimentation.

### L15 EPS enhances osteogenic differentiation of hDPSCs

**Fig. 4:**
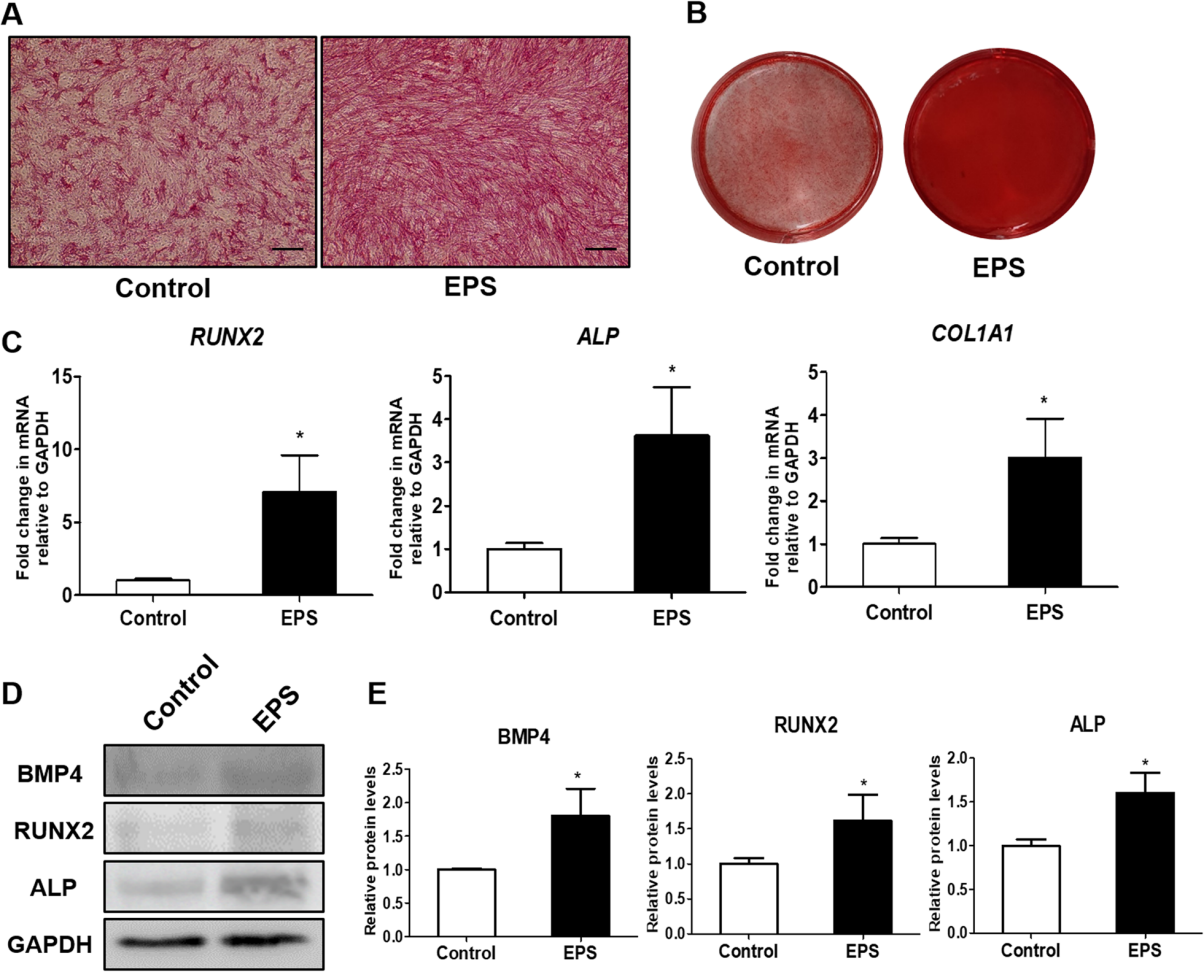
L15 EPS enhances osteogenic differentiation of hDPSCs. **A** Photomicrograph of ALP staining of hDPSCs with or without L15 EPS. Inverted imaging system (EVOS™ XL Core Imaging System; Thermo Scientific™, Waltham, MA, USA). Scale bar = 200 μm **B** Photograph showing calcium deposition of hDPSCs with or without L15 EPS by ARS staining. **C** The expression of osteogenic differentiation marker genes (*RUNX2, ALP,* and *COL1A1*) were analyzed by real time-PCR. **D** The expression of osteogenic differentiation marker proteins (BMP4, RUNX2, and ALP) expression were analyzed by western blot analysis. (E) Quantitative analysis of intensity in (D) relative to GAPDH. Scale bar = 200 μm. *p < 0.05, *N* = 3

ALP staining was performed after treatment of L15 EPS for 7 days. ALP staining was more intense in the L15 EPS treatment group than in the control group (Fig. 4A). The calcium accumulation was examined by ARS staining after 28 days of osteogenic differentiation. The calcium deposits were significantly increased in the L15 EPS treatment group compared to the control group (Fig. 4B). The expression levels of the osteogenesis-related genes, *RUNX2*, *ALP*, and *COL1A1* were significantly up-regulated in the L15 EPS treatment group compared to the control group (Fig. 4C). The protein expression levels of BMP4, RUNX2, and ALP were significantly increased in the L15 EPS treated (Fig. 4D, E; *p* < 0.05).

### L15 EPS promotes osteogenic differentiation via the p38 MAPK pathway

**Fig. 5:**
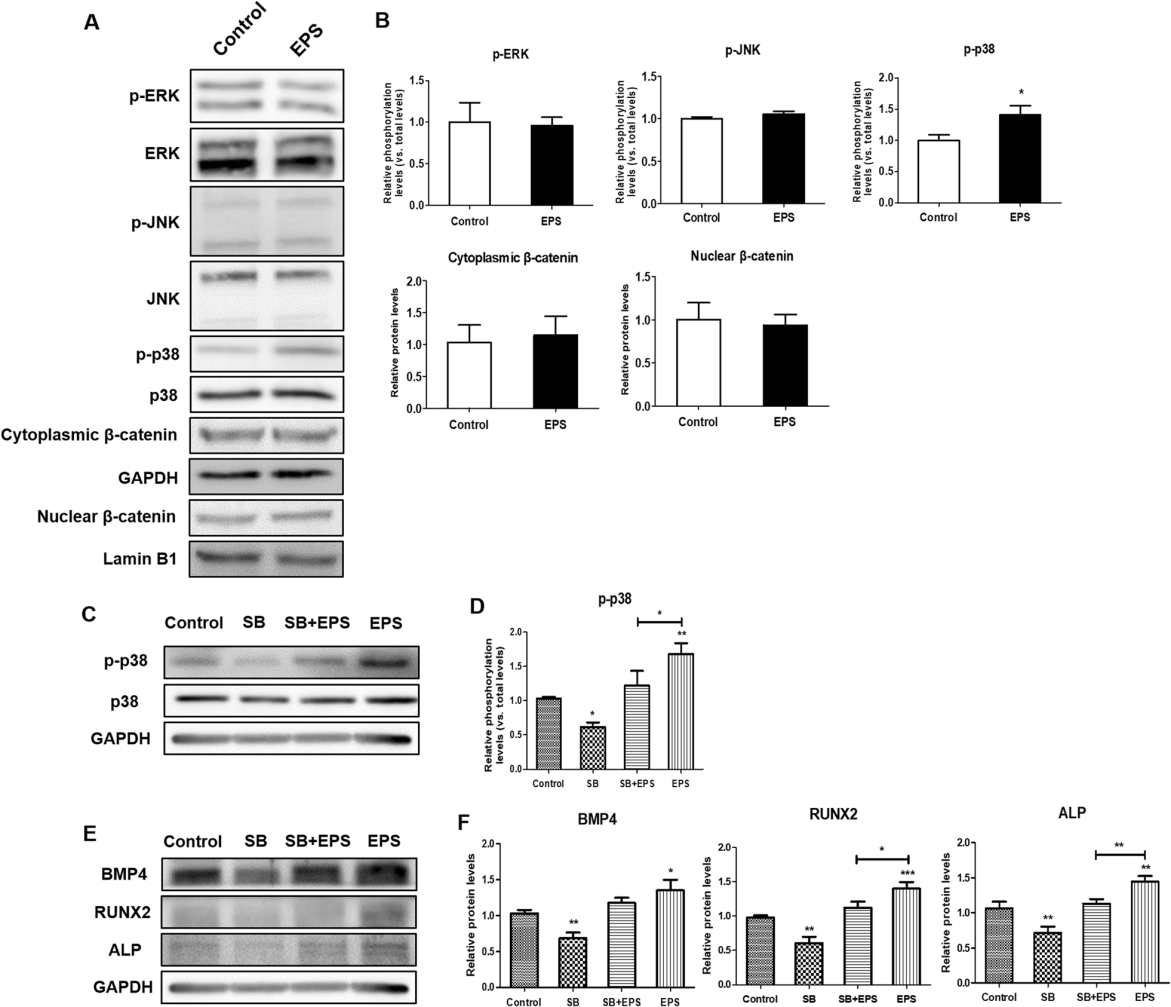
The L15 EPS activated p38 MAPK pathways. **A** Western blot analysis of MAPK (ERK, JNK, and p38) and Wnt signal pathways in hDPSCs treated with L15 EPS. **B** Quantitative analysis of phosphorylation levels, cytoplasmic and nuclear β-catenin levels in **A**. **C** Western blot analysis for proteins p38 and p-p38 levels after inhibition of the p38 MAPK pathway. **D** Quantitative analysis of intensity in **C**. **E** Western blot analysis for osteogenic differentiation marker proteins (BMP4, RUNX2, and ALP) levels after inhibition of the p38 MAPK pathway. **F** Quantitative analysis of intensity in **E**. *p < 0.05, **p < 0.01, ***p < 0.001. *N* = 3

The effect of L15 EPS on the levels of pathway proteins (i.e., ERK, p-ERK, JNK, p-JNK, p38, p-p38, cytoplasmic β-catenin and nuclear β-catenin) were verified by western blot analysis (Fig. 5A). Since nuclear translocation of β-catenin is a key feature of Wnt pathway activation, nuclear and cytoplasmic fractionation of β-catenin was conducted. The expression levels of ERK, p-ERK, JNK, p-JNK, cytoplasmic β-catenin and nuclear β-catenin did not change (Fig. 5B). The expression of p-p38 was upregulated in the L15 EPS treatment group (Fig. 5B). The ratio of p-p38/p38 was greatly increased in the L15 EPS treatment group compared to the control group (Fig. 5B). These results showed that the p38 MAPK pathway was activated after L15 EPS treatment.

### Inhibition of p38 MAPK pathways reduced L15 EPS-induced osteogenic differentiation of hDPSCs

The role of p38 in L15 EPS-induced osteogenic differentiation of hDPSCs was further analyzed using the p38 inhibitor SB203580 (SB). The efficiency of the SB was confirmed by western blot (Fig. 5C). SB led to a remarkable decrease in the ratio of p-p38/p38 (Fig. 5D). The ratio of p-p38/p38 was markedly upregulated in the EPS treatment group compared to the control group (Fig. 5D). Quantitative analysis showed that the ratio of p-p38/p38 in SB + EPS treatment group was inhibited (Fig. 5D). After osteogenic differentiation for 14 days, RUNX2 and ALP protein expression levels were significantly downregulated in SB + EPS treatment group compared to the EPS treatment group (Fig. 5E, F).

### The effect of L15 EPS on osteoblast precursors from calvaria

**Fig. 6:**
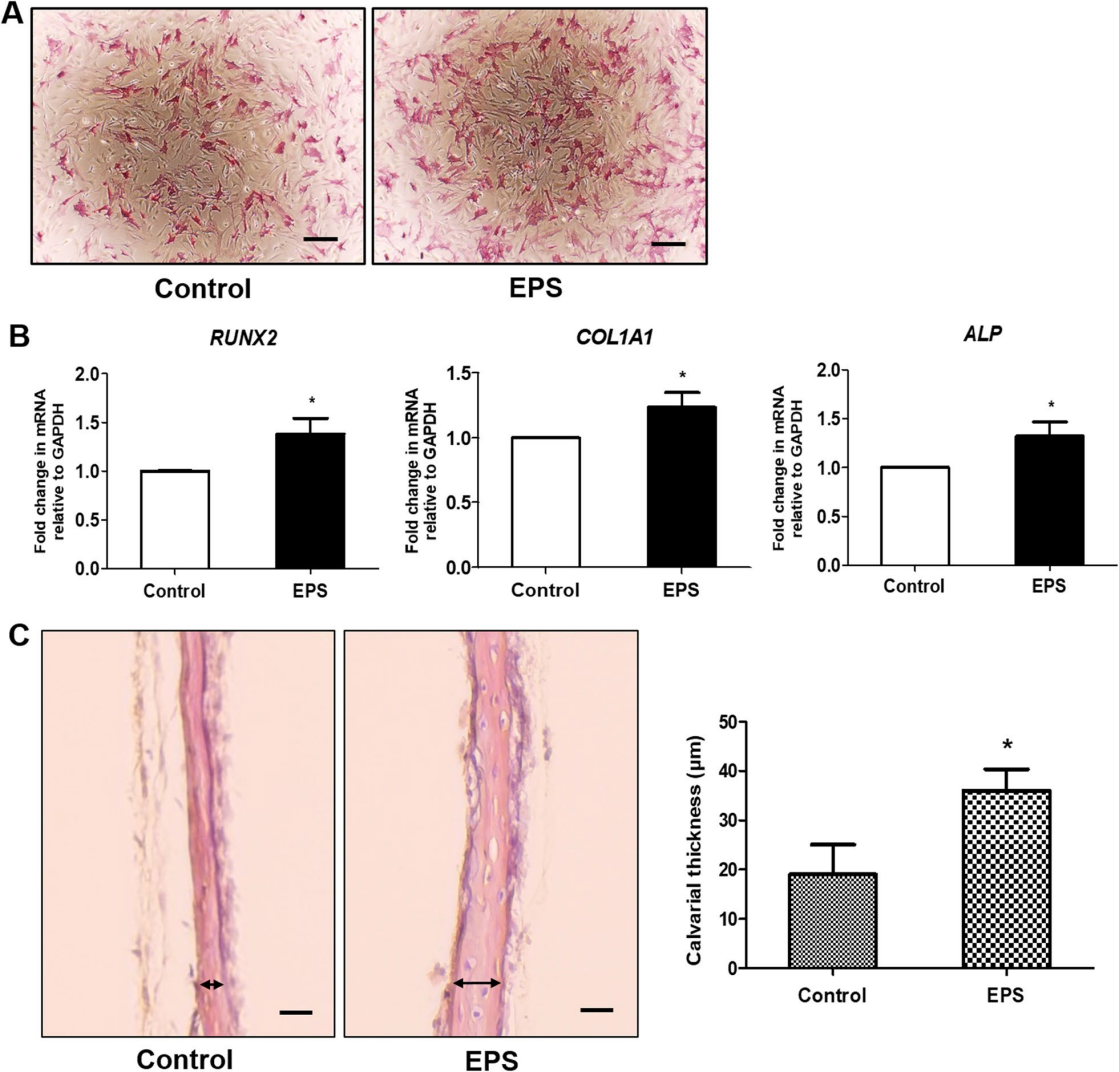
The effect of L15 EPS on primary osteoblast precursors and bone formation. **A** Photomicrographs of ALP staining of primary osteoblast precursor obtained from the calvaria of ICR neonates with or without L15 EPS for 7 days. Inverted imaging system (EVOS™ XL Core Imaging System; Thermo Scientific™, Waltham, MA, USA). Scale bar = 200 μm. **B** The expression of osteogenic differentiation marker genes (*RUNX2, COL1A1,* and *ALP*) in primary osteoblast precursor were analyzed by real time-PCR. **C** The ICR neonates calvarial ex vivo organ culture was performed with or without L15 EPS. Calvaria was cultured for 7 days and then processed for histological examination. Thickness of the calvaria was assessed by H&E staining (Olympus BX50 microscope; Scale bar = 25 μm). *p < 0.05. *N* = 3

To determine whether L15 EPS improves osteogenic differentiation of osteoblast precursors, isolated osteoblast precursors were cultured in osteogenic medium with or without L15 EPS for 7 days. ALP intensity was stronger in L15 EPS treatment group relative to the control group (Fig. 6A). After 14 days of osteogenic differentiation with L15 EPS, mRNA levels were analyzed by qPCR. The mRNA levels of *RUNX2*, *COL1A1*, and *ALP* were significantly upregulated with L15 EPS (Fig. 6B).

### L15 EPS increases the bone formation in calvarial organ culture model

The effect of L15 EPS on bone formation was confirmed using calvarial ex vivo organ culture. H&E staining showed that L15 EPS increased thickness of bone (Fig. 6C). These results indicate that L15 EPS can induce bone formation.

## Discussion

Probiotics consisting of cellular components and metabolites can show biological activities, including immunomodulatory, antimicrobial, and antioxidant effects [48–50]. Interest in postbiotics and parabiotics as alternatives to live probiotics has increased due to their enhanced safety, easier storage, and longer shelf life [50]. Microbial polysaccharides, which are macromolecular metabolites made of many smaller monosaccharides, include capsular polysaccharides, EPSs, lipopolysaccharides (LPSs), and peptidoglycans [51]. Several reports have shown positive effects of microbial and botanical polysaccharides on osteogenic differentiation. Specifically, the combination of LPS and PLLA nanofibers has been shown to promote the osteogenic differentiation of MSCs [52], and plant astragalus polysaccharide was shown to enhance the osteogenic differentiation of BMSCs [53]. LPS is present only in gram-negative bacteria, whereas *E. faecium,* gram-positive bacterium, has no LPS. Osteogenic differentiation potential on hDPSCs was tested in this study initially with L15 extracts and later with their EPSs.

RUNX2 is an essential transcription factor in osteogenic differentiation [54, 55], and it is involved in the production of BMPs [56]. Moreover, autocrine BMP production is necessary for RUNX2 to actively facilitate osteogenic differentiation [57]. Both the MAPK and Wnt/β-catenin pathways play a crucial role in osteogenic differentiation [58–61]. MAPKs are a family of enzymes composed of extracellular signal-regulated kinases (ERKs), c-Jun amino-terminal kinases (JNKs), and p38 MAPK [62]. Both ERK and p38 MAPKs are crucial in RUNX2 activation and osteogenic differentiation [59–61], while the role of JNK is controversial; for instance, the activation of JNK induced osteoblastic differentiation of human periosteal-derived cells [63]. The inhibition of JNK in human mesenchymal stem cells increased osteogenic differentiation marker expression [64], which complicates our understanding of the role of JNK. To identify the osteogenic differentiation mechanism of L15 EPS, the present study detected the expression of β-catenin and the phosphorylation of ERK, JNK, and p38 MAPK by western blot. L15 EPS, upregulated the expression of p-p38 in hDPSCs, suggests that the p38 MAPK pathway was activated in hDPSCs during osteogenic differentiation. To verify the function of p38 MAPK signaling in osteogenic differentiation of hDPSCs, p38 pathway inhibitor SB was applied. Inhibition of the p38 pathway suppressed L15 EPS induced-osteogenic differentiation of hDPSCs. These findings suggest that L15 EPS promotes osteogenic differentiation of hDPSCs via the p38 MAPK pathway. Furthermore, this study demonstrated the increased osteogenic differentiation of osteoblast precursors and thickened the bone of mouse calvaria.

Bone defects and bone-related diseases do not directly impact human survival, but they can severely affect quality of life. Bone regeneration remains an important challenge in medicine, especially orthopedics, and oral/maxillofacial surgery. Through biomimetics, novel materials and scaffolds for bone tissue regeneration have been developed [2, 65–67]. Bone tissue engineering involves multiple steps. Cells with osteoinductive molecules are incorporated into scaffolds and pre-incubated in vitro, followed by the cell-loaded scaffolds being implanted into the area of bone defect [68]. In this step, it is important to choose the right cells and osteoinductive molecules. DPSCs have been considered as an alternative cell source of mesenchymal stem cells for tissue repair [69, 70]. It has reported that DPSC loaded scaffolds are capable of bone regeneration [4, 71]. Since this study has shown that *E. faecium* L15 EPS promotes osteogenic differentiation of DPSCs, DPSCs and L15 EPS may be useful for the treatment of bone regeneration.

In summary, this study demonstrated that L15 EPS enhanced osteogenic differentiation of hDPSCs by activating the p38 MAPK pathway to promote bone formation. This is the first study to apply postbiotic EPS to induce osteogenic differentiation. The findings confirmed a beneficial effect of *E. faecium* L15 extract and L15 EPS on osteogenic differentiation, while revealing the mechanisms involved in postbiotic biofunctional effects. Further studies should be pursued to identify the chemical structure of L15 EPS and to explore the effect of L15 EPS-mediated bone regeneration in animal models.

## Conclusions

In this study, we investigated that the influence of the *E. faecium* L15 extract and EPS on osteogenic differentiation of hDPSCs and the underlying mechanism. The L15 extract and EPS promoted the osteogenic differentiation of hDPSCs as demonstrated by increasing expression levels of osteogenesis-related markers and calcium deposition. The involvement of p38 MAPK signaling on osteogenic differentiation of hDPSCs was examined using p38 inhibitor, SB203580. As a result, the L15 EPS enhances the osteogenic differentiation of hDPSCs through the p38 MAPK pathway. The effect of promoting osteogenic differentiation of L15 EPS was also observed in osteoblast precursors. Moreover, L15 EPS boosted bone formation in ex vivo organ culture. This is the first evidence of the osteogenic differentiation effect exerted by EPS from LAB (*E. faecium* L15 EPS). As demonstrated by the present study, L15 EPS may have therapeutic value in bone regeneration as potential postbiotics. As a follow-up study, a comprehensive chemical characterization of L15 EPS, together with relevant animal research, should be pursued.

## Supplementary Information

**Additional file 1.** Uncropped western blot images. Uncropped western blot images are attached. Images used in the main figure are marked in red squares.

## Data Availability

All data generated or analyzed during this study are included in this published article and its Additional file 1.

## Abbreviations

EPS: Exopolysaccharide
hDPSC: Human dental pulp stem cell
ALP: Alkaline phosphatase
MAPK: Mitogen-activated protein kinase
LAB: Lactic acid bacteria
PBS: Phosphate-buffered saline
FBS: Fetal bovine serum
FACS: Fluorescence-activated cell sorting
FITC: Fluorescein isothiocyanate
APC: Allophycocyanin
WST: Water-soluble tetrazolium salt
RT-PCR: Reverse transcription polymerase chain reaction
GAPDH: Glyceraldehyde 3-phosphate dehydrogenase
ARS: Alizarin red S
H&E: Hematoxylin and eosin
ANOVA: A one-way analysis of variance
COL1A1: Collagen type I
BMP4: Bone morphogenetic protein-4
LPS: Lipopolysaccharide
ERK: Extracellular signal regulated kinase
JNK: C-Jun amino-terminal kinase
