# Supplementary material for: Exopolysaccharide of Enterococcus faecium L15 promotes the osteogenic differentiation of human dental pulp stem cells via p38 MAPK pathway
Source: Stem Cell Res Ther. 2022 Sep 2;13:446. doi: 10.1186/s13287-022-03151-0 (PMC9440579; doi:10.1186/s13287-022-03151-0)

Exopolysaccharide of *Enterococcus faecium* L15 promotes the osteogenic differentiation of human dental pulp stem cells via p38 MAPK pathway

Hyewon Kim[1], Naeun Oh[1], Mijin Kwon[1], Oh-Hee Kwon[2], Seockmo Ku[3], Jeongmin Seo[1,2,*] and Sangho Roh[1,*]

[1] Cellular Reprogramming and Embryo Biotechnology Laboratory, Dental Research Institute, Seoul National University School of Dentistry, Seoul 08826, Korea; 201520496@snu.ac.kr (H.K.); onajun@naver.com (N.O.); rnjsalwls23@hanmail.net (M.K.); sangho@snu.ac.kr (S.R.)

[2] Biomedical Research Institute, NeoRegen Biotech Co., Ltd., Gyeonggi-do 16641, Korea; ohee@neoregenbio.com (O-H.K.); jminseo@gmail.com (J.S.)

[3] Fermentation Science Program, School of Agriculture, College of Basic and Applied Sciences, Middle Tennessee State University, Murfreesboro, TN 37132, USA; seockmo.ku@mtsu.edu (S.K.)

* Corresponding author: jminseo@gamil.com (J.S.); sangho@snu.ac.kr (S.R.); Tel.:+82-2-880-2333 (J.S. & S.R.)

Supplementary figure 1. Uncropped western blot images of figure 2

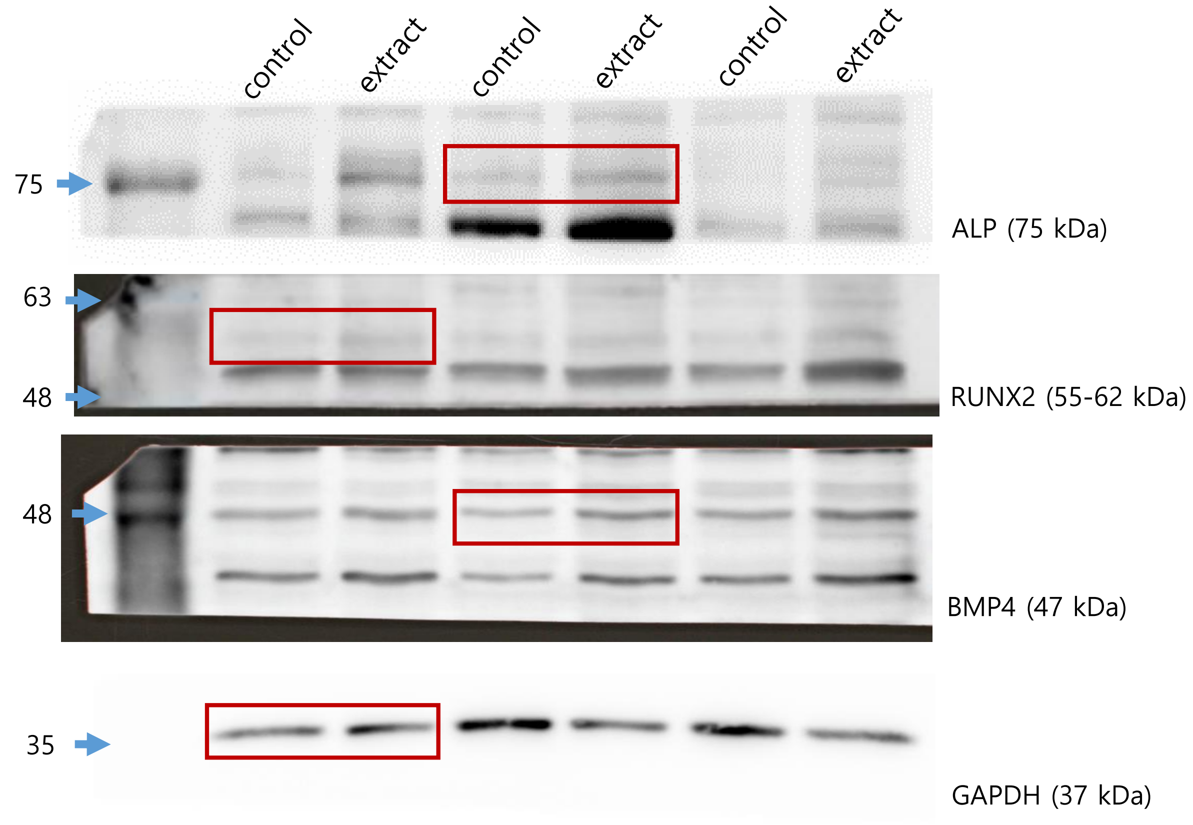

Supplementary figure 2. Uncropped western blot images of figure 4

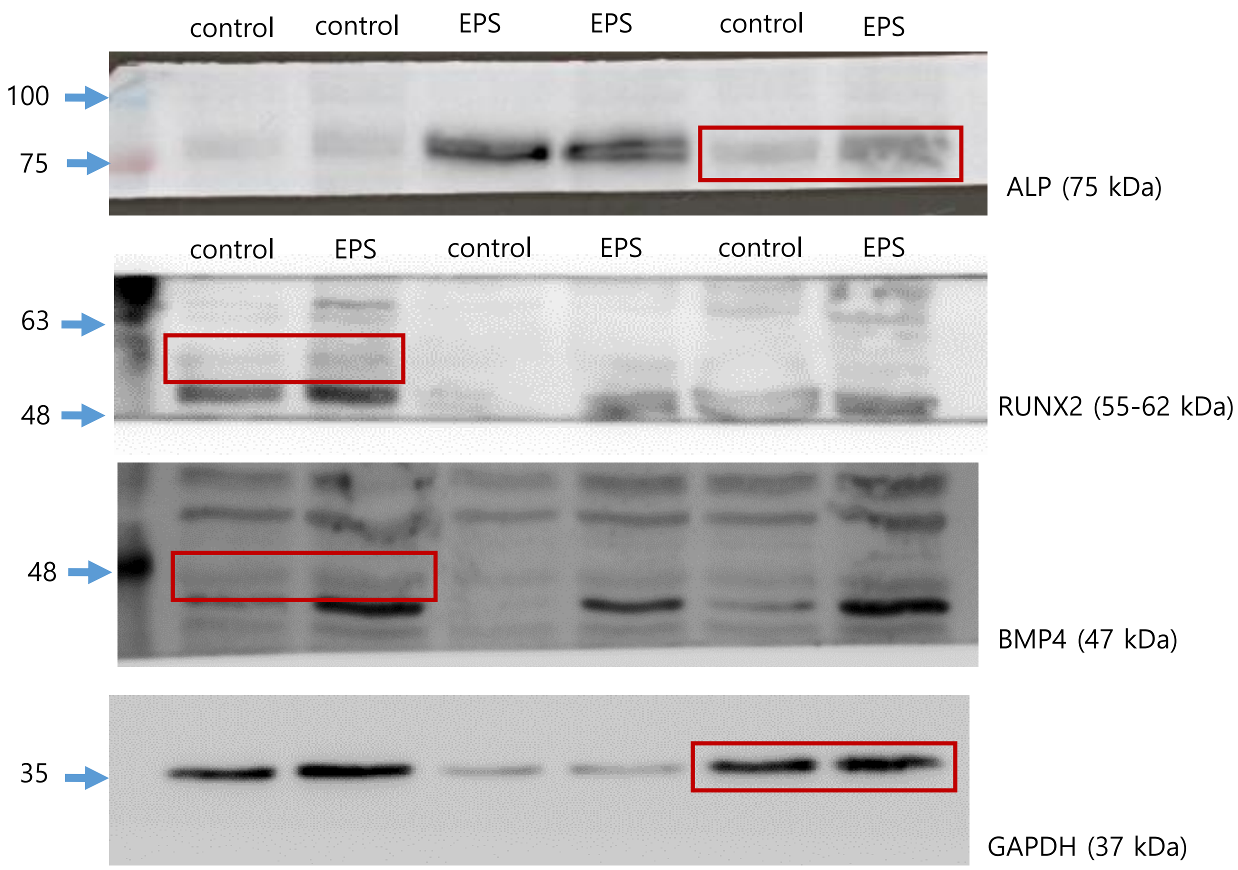

Supplementary figure 3. Uncropped western blot images of figure 5

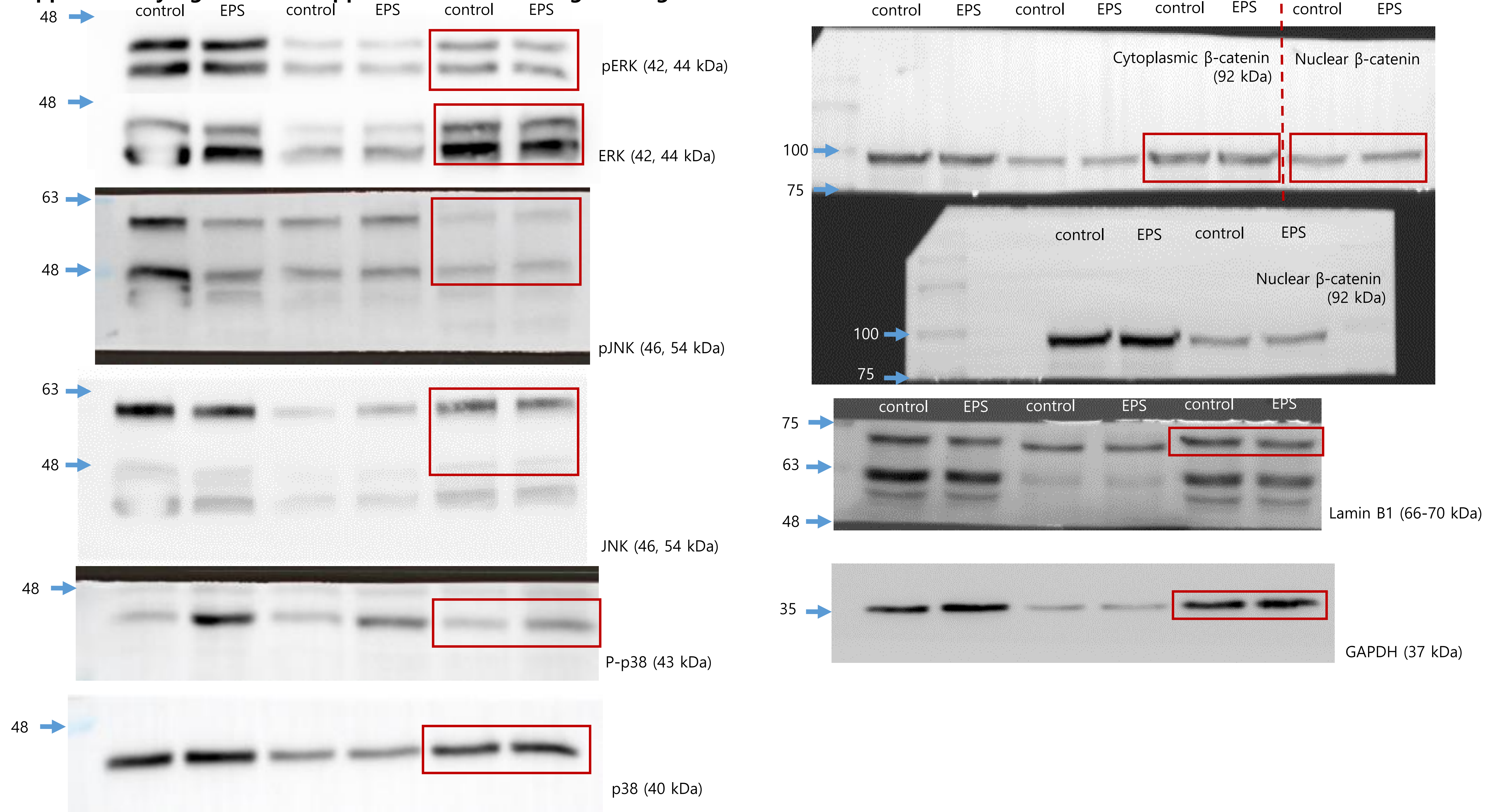

Supplementary figure 4. Uncropped western blot images of figure 5

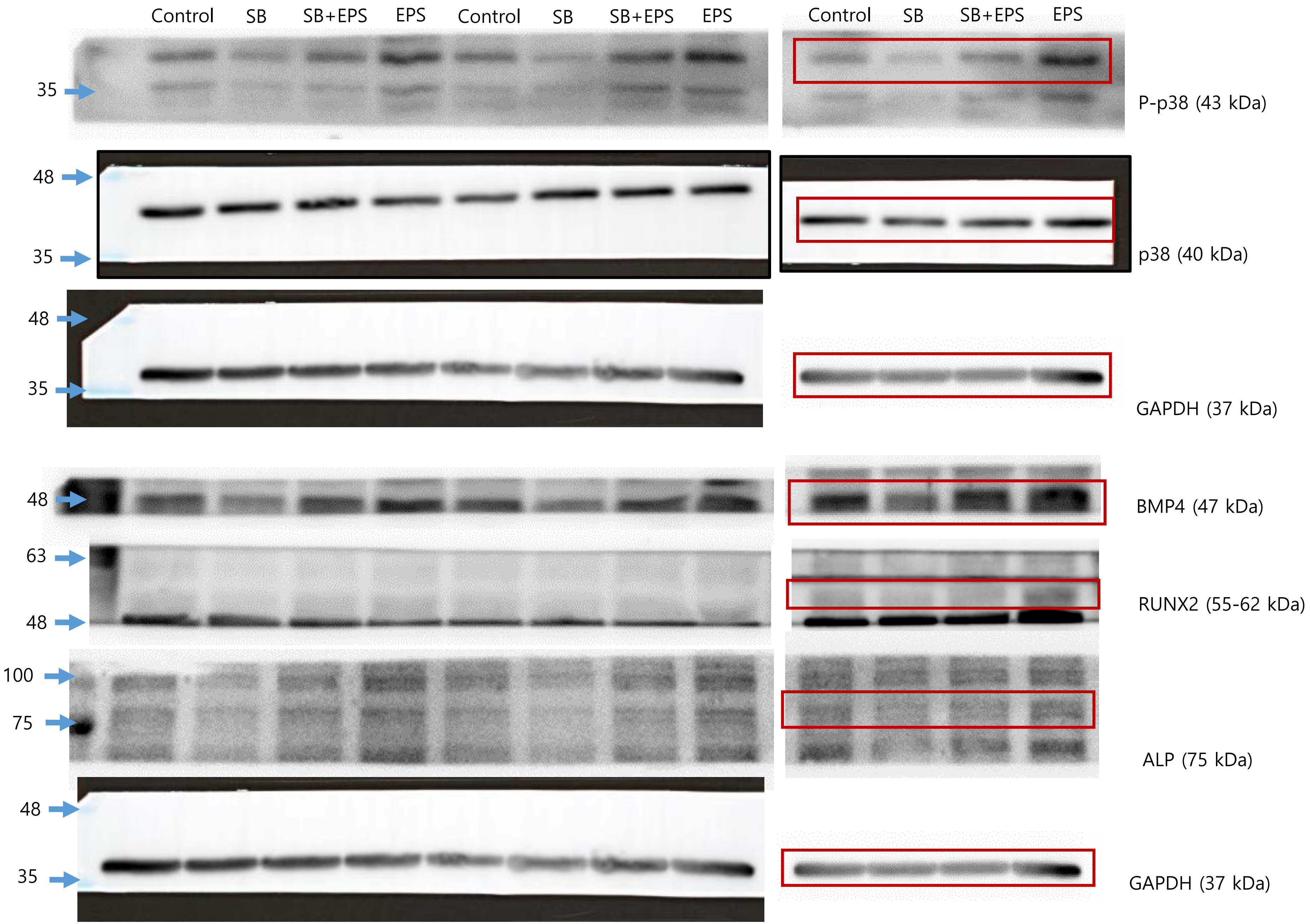

Supplement: Supplementary file 1 — Additional file 1. Uncropped western blot images. Uncropped western blot images are attached. Images used in the main figure are marked in red squares. [file 13287_2022_3151_MOESM1_ESM.pdf]
